# Influence of statin use on the incidence of recurrent venous thromboembolism and major bleeding in patients receiving rivaroxaban or standard anticoagulant therapy

**DOI:** 10.1186/1477-9560-12-26

**Published:** 2014-11-26

**Authors:** Philip S Wells, Martin Gebel, Martin H Prins, Bruce L Davidson, Anthonie WA Lensing

**Affiliations:** Department of Medicine, University of Ottawa, Ottawa Hospital Research Institute, Ottawa, Canada; Bayer HealthCare, Wuppertal, Germany; Maastricht University Medical Center, Maastricht, The Netherlands; University of Washington School of Medicine, Seattle, WA USA

**Keywords:** Anticoagulant therapy, Rivaroxaban, Statins, Venous thromboembolism

## Abstract

**Background:**

Statins may reduce the risk of first and recurrent venous thromboembolism (VTE). No data are available on their potential benefit in patients treated with the oral anticoagulant rivaroxaban.

**Methods:**

The EINSTEIN DVT/PE and EINSTEIN Extension studies compared rivaroxaban with standard of care (n=8280) and placebo (n=1188), respectively. The incidences of recurrent VTE and major bleeding per 100 patient-years for exposure (or not) to statins were calculated. A Cox proportional hazards model was constructed, stratified by index event and intended treatment duration, with statin use as a time-dependent variable, for each treatment group (rivaroxaban vs enoxaparin/vitamin K antagonist or placebo) and adjusted for relevant variables.

**Results:**

In EINSTEIN DVT/PE, 1509 (18.3%) patients used statins during the at-risk period, and 6731 (81.7%) did not. Overall, 2.6 recurrent VTEs occurred per 100 patient-years with statin use compared with 3.8 per 100 patient-years without statins (adjusted hazard ratio [HR] 0.76; 95% confidence interval [CI] 0.46–1.25). HRs for recurrent VTE were similar for concomitant use of rivaroxaban-statin and enoxaparin/VKA-statin. Major bleeding events occurred in 3.0 per 100 patient-years with statin use compared with 2.3 per 100 patient-years without statins (adjusted HR 0.77; 95% CI 0.46–1.29). Due to adjustments in the Cox regression model, the direction of this HR is in contrast to the crude comparison. In EINSTEIN Extension, no recurrent VTEs occurred with statin use in the rivaroxaban group compared with 1.6 per 100 patient-years without statins. In the placebo group, 12.2 recurrent VTEs occurred per 100 patient-years with statin use compared with 13.2 per 100 patient-years without (adjusted HR 0.81; 95% CI 0.35–1.86).

**Conclusions:**

The effect of statins in this secondary analysis of the EINSTEIN VTE treatment program is consistent with other studies that suggest a reduced risk of recurrent VTE, but conclusive evidence of this benefit is lacking. Statins are simple to use, inexpensive, very safe and do not cause bleeding. Therefore, the potential effect on reducing recurrent VTE, which is in the range of that of acetylsalicylic acid, deserves evaluation in a large randomized trial.

**Trial registration number:**

ClinicalTrials.gov: EINSTEIN PE, NCT00439777; EINSTEIN DVT, NCT00440193; EINSTEIN Extension, NCT00439725.

## Background

Statins are prescribed primarily with the aim of lowering levels of low-density lipoprotein cholesterol in patients at increased risk for cardiovascular events. In recent years, there has been an interest in the potential of statins to reduce the risk of venous thromboembolism (VTE), comprising deep vein thrombosis (DVT) and pulmonary embolism (PE), driven by the publication of several studies and meta-analyses suggesting a significant protective effect [1–3]. However, the most recent meta-analysis showed an odds ratio (OR) for a first venous thromboembolic event for statin users of only 0.89 (95% confidence interval [CI] 0.78–1.01) compared with non-users [4]. Recently, registry based studies suggested that statins could also diminish the rate of recurrent VTE [5–7].

Anticoagulant therapy is the standard approach for the treatment of VTE. In the large phase III EINSTEIN DVT and PE program, the oral, direct Factor Xa inhibitor rivaroxaban was compared with parenteral enoxaparin and oral vitamin K antagonist (VKA) for the acute treatment of VTE [8–10], and with placebo for the extended treatment of VTE [8]. A relatively high proportion of patients enrolled in these studies were receiving concomitant medication with statins. This post-hoc analysis evaluated outcomes in terms of recurrent VTE and major bleeding during the period patients received or did not receive statins.

## Methods

### Patients and design

The methodology of the EINSTEIN DVT and PE program has been described previously [8–10]. Briefly, more than 8000 patients with acute symptomatic, confirmed DVT and/or symptomatic, confirmed and hemodynamically stable PE were randomized to receive either fixed-dosed rivaroxaban (15 mg twice-daily for 3 weeks followed by 20 mg once-daily) or standard treatment with enoxaparin overlapping with and transitioning to VKA, adjusted to maintain an international normalized ratio between 2.0 and 3.0. Treatment was continued for 3, 6 or 12 months. For the extended treatment of VTE, more than 1100 patients who had completed a 6–12 month course of anticoagulant treatment were randomized to rivaroxaban 20 mg or placebo for an extra 6 or 12 month period [8].

Ethical approval was obtained from the Institutional Review Boards of all institutions involved in the EINSTEIN studies, and written informed consent was provided by all patients [10].

The main exclusion criteria were another indication for a VKA; a calculated creatinine clearance <30 ml/min using the Cockcroft–Gault formula; clinically significant liver disease or an alanine aminotransferase level that was three times the upper limit of the normal range or higher; active bleeding or a high risk of bleeding, contraindicating anticoagulant treatment; systolic blood pressure greater than 180 mm Hg or diastolic blood pressure greater than 110 mm Hg; childbearing potential without proper contraceptive measures, pregnancy, or breast-feeding; concomitant use of strong cytochrome P450 3A4 inhibitors (e.g. human immunodeficiency virus protease inhibitors or systemic ketoconazole) or inducers (e.g. rifampicin, carbamazepine or phenytoin); and a life expectancy of less than 3 months. The protocols did not include guidelines for the use of statins.

Patients were followed clinically and in case of a suspected recurrent DVT/PE, a work-up using objective diagnostic tests was required. Symptomatic recurrent VTE was defined as a composite of fatal or non-fatal PE or DVT on the basis of criteria that have been described previously [8, 9]. Death was classified as due to PE, bleeding, or other established causes or diagnoses. PE was considered the cause of death if there was objective documentation of the condition or if death could not be attributed to a documented cause and PE could not be confidently ruled out. Bleeding was defined as major if it was clinically overt and associated with a decrease in hemoglobin of ≥2.0 g/dl; led to the transfusion of ≥2 units of red blood cells; was intracranial, retroperitoneal or occurred in another critical site; or contributed to death [8, 9]. All suspected outcome events were classified by a central adjudication committee whose members were unaware of the treatment assignment.

### Analysis

Concomitant medications taken during the studies were collected at each visit on an electronic case record form, and 100% source verification was done by a clinical research associate. Statin therapies were coded according to the WHO Drug Dictionary Version 2005/Q3 and were defined via the Anatomical-Therapeutic-Chemical (ATC) codes. Statins were classified as low, medium or high potency based on their low-density lipoprotein reduction capacity, as previously described [7].

Analyses were performed in SAS version 9.2 (SAS Institute Inc., Cary, NC, USA). The use of statin therapy was categorized as either ‘on’ or ‘off’ for each day between the day of randomization and the end of the patient’s at-risk period, which was defined as last intake of rivaroxaban or enoxaparin/VKA (or placebo) plus 2 days, or the onset date of 1) the first confirmed recurrent DVT/PE, or 2) major bleeding, if these occurred earlier. For the extended treatment study, major bleeding was not analyzed, because only four major bleeding events occurred [8]. Person-time was accumulated per patient from the day of randomization until the end of the at-risk period. Patients who were ‘on’ and ‘off’ statin therapy during the at-risk period contributed person-time to both (‘on’ and ‘off’) categories of exposure. The incidence densities for the primary efficacy outcome and major bleeding were expressed as incidence rates per 100 patient-years of exposure (or not) to statins, both overall and by age group (<60 years or ≥60 years) for statin users and non-users in both treatment arms combined and separately. A Cox proportional hazards model was constructed, stratified by index event and intended treatment duration, with statin use as a time-dependent variable, for each treatment group (rivaroxaban vs enoxaparin/VKA or placebo) and adjusted for acetylsalicylic acid (ASA) use, age (<60 years vs ≥60 years), body mass index (≥30 kg/m^2^ vs <30 kg/m^2^), cardiac disorders, sex, creatinine clearance at baseline (≥50 ml/min vs <50 ml/min), and interaction between treatment group and statin use. P-values for interaction were calculated. Separate models including interaction between each covariate and statin use were constructed to generate hazard ratios for use versus non-use by category as well as p-values for interaction. Due to zero cell counts, the Cox proportional hazards model for the extended treatment study was only adjusted for age and prior rivaroxaban or VKA treatment. A Poisson regression was used to calculate the interaction p-value for potency and treatment group.

## Results

### Patient demographics

Baseline demographic characteristics are summarized in Table 1 for the acute EINSTEIN DVT and PE program and in Table 2 for the extended rivaroxaban treatment study. In total, 1509 (18.3%) patients included in the acute EINSTEIN DVT or PE studies used statins (simvastatin, n=716; atorvastatin, n=532; rosuvastatin, n=171; others, n=164) and during the at-risk period, and 6731 (81.7%) did not. A total of 1223 (81.0%) patients used statins throughout the-at-risk period. In the extended treatment study, a total of 230 (19.4%) patients used statins during the at-risk period and 958 (80.6%) did not. In all studies, statin users were older, presented more often with PE, had higher frequencies of renal impairment and cardiovascular disorders, and used ASA more often [11].

**Table 1:** **Baseline characteristics of patients with and without statin therapy for rivaroxaban and enoxaparin/VKA combined**

Characteristic	Patients treated with statins (n=1509)	Patients not treated with statins (n=6731)	p-value
Mean age, years	66.5	54.9	<0.01
Men, n (%)	847 (56.1)	3650 (54.2)	0.18
Mean BMI, kg/m^2^	29.3	27.8	<0.01
Creatinine clearance, n (%)			<0.01
<50 ml/min	207 (13.7)	442 (6.6)	
50–<80 ml/min	515 (34.1)	1500 (22.3)	
≥80 ml/min	775 (51.4)	4736 (70.4)	
Missing	12 (0.8)	53 (0.8)	
Planned treatment duration, n (%)			<0.01
3 months	75 (5.0)	580 (8.6)	
6 months	858 (56.9)	4056 (60.3)	
12 months	576 (38.2)	2095 (31.1)	
Index event, n (%)			<0.01
Only DVT	489 (32.4)	2880 (42.8)	
PE ± DVT	1012 (67.1)	3783 (57.2)	
Index event not confirmed or evaluable	8 (0.5)	68 (1.0)	
Immobilization at randomization, n (%)	232 (15.4)	1051 (15.6)	0.82
Active cancer at randomization, n (%)	76 (5.0)	352 (5.2)	0.76
Ischemic heart disease, n (%)	410 (27.2)	229 (3.4)	<0.01
Peripheral arterial disease, n (%)	39 (2.6)	29 (0.4)	<0.01
Ischemic cerebrovascular disease, n (%)	53 (3.5)	45 (0.7)	<0.01
ASA use at baseline, n (%)	387 (26.5)	337 (5.0)	<0.01
ASA stopped at randomization, n	85	122	
Hypertension, n (%)	1052 (69.7)	2181 (32.4)	<0.01
Diabetes, n (%)	392 (26.0)	512 (7.6)	<0.01

ASA, acetylsalicylic acid; BMI, body mass index; DVT, deep vein thrombosis; PE, pulmonary embolism; VKA, vitamin K antagonist.

Data from EINSTEIN DVT and EINSTEIN PE combined, safety population.

**Table 2:** **Baseline characteristics with/without statin therapy for patients allocated to rivaroxaban and placebo (shown separately)**

	Rivaroxaban		p-value	Placebo		p-value
	Patients treated with statins (n=113)	Patients not treated with statins (n=485)		Patients treated with statins (n=117)	Patients not treated with statins (n=473)	
Mean age, years	64.3	56.7	<0.01	66.9	56.3	<0.01
Men, n (%)	62 (54.9)	289 (59.6)	0.36	66 (56.4)	272 (57.5)	0.42
Mean BMI, kg/m^2^	29.9	28.1	<0.01	29.8	28.0	<0.01
Creatinine clearance, n (%)			0.20			<0.01
<50 ml/min	10 (8.8)	31 (6.4)		17 (14.5)	33 (7.0)	
50–<80 ml/min	33 (29.2)	113 (23.3)		39 (33.3)	96 (20.3)	
≥80 ml/min	68 (60.2)	335 (69.1)		60 (51.3)	340 (71.9)	
Missing	2 (1.8)	6 (1.2)		1 (0.9)	4 (0.8)	
Planned treatment duration, n (%)			0.14			0.19
6 months	61 (54.0)	298 (61.4)		68 (58.1)	285 (60.3)	
12 months	52 (46.0)	187 (38.6)		49 (41.9)	188 (39.7)	
Index event, n (%)			<0.01			<0.01
Only DVT	48 (42.5)	325 (67.0)		54 (46.2)	292 (61.7)	
PE ± DVT	61 (54.0)	151 (31.1)		61 (52.1)	172 (36.4)	
Index event not confirmed	4 (3.5)	9 (1.9)		2 (1.7)	9 (1.9)	
Immobilization at randomization, n (%)	17 (15.0)	72 (14.8)	0.96	17 (14.5)	59 (12.5)	0.66
Active cancer at randomization, n (%)	5 (4.4)	23 (4.7)	0.89	6 (5.1)	20 (4.2)	0.85
Ischemic heart disease, n (%)	19 (16.8)	14 (2.9)	<0.01	40 (34.2)	20 (4.2)	<0.01
Peripheral arterial disease, n (%)	1 (0.9)	2 (0.4)		1 (0.9)	0	
Ischemic cerebrovascular disease, n (%)	0	4 (0.8)		2 (1.7)	5 (1.1)	
ASA use at baseline, n (%)	5 (4.4)	15 (3.1)	<0.01	29 (24.8)	18 (3.8)	<0.01
ASA stopped at randomization, n	0	3		0	0	
Hypertension, n (%)	80 (70.8)	161 (33.2)	<0.01	73 (62.4)	154 (32.6)	<0.01
Diabetes, n (%)	30 (26.5)	28 (5.8)	<0.01	35 (29.9)	21 (4.4)	<0.01

ASA, acetylsalicylic acid; BMI, body mass index; DVT, deep vein thrombosis; PE, pulmonary embolism.

Data from EINSTEIN Extension, safety population.

### Acute EINSTEIN DVT and PE studies

#### Recurrent venous thromboembolism

**Figure 1:**
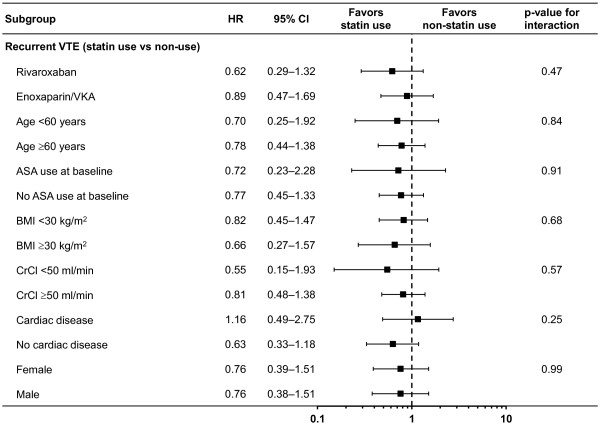
**Hazard ratio and 95% CIs for recurrent VTE by statin use versus no statin use.** Important subgroups of patients in the EINSTEIN DVT and PE studies. ASA, acetylsalicylic acid; BMI, body mass index; CI, confidence interval; CrCl, creatinine clearance; HR, hazard ratio; VKA, vitamin K antagonist; VTE, venous thromboembolism.

For the treatment groups combined, 20 recurrent VTEs occurred during 783.8 patient-years (2.6 per 100 patient-years) with statin use compared with 146 during 3872.1 patient-years (3.8 per 100 patient-years) without use of statins, for an adjusted hazard ratio [HR] of 0.76 (95% CI 0.46–1.25). Hazard ratios for recurrent VTE were similar for concomitant use of rivaroxaban-statin and enoxaparin/VKA-statin (p_interaction_=0.47, Figure 1). Hazard ratios for recurrent VTE with the use of statins were similar in all other important subgroups of patients (Figure 1).

Recurrent VTE occurred in 1 (3.0%) per 100 patient-years in the group of patients that used a low potency statin, in 14 (2.3%) per 100 patient-years in the group of patients with a medium potency statin, and in 4 (4.1%) per 100 patient-years in the group of patients with a high potency statin. The respective rates were similar for rivaroxaban and enoxaparin/VKA patients (p_interaction_=0.84).

#### Major bleeding

**Figure 2:**
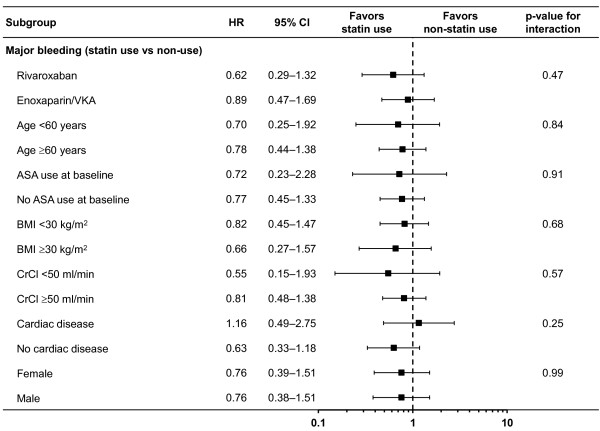
**Hazard ratio and 95% CIs for major bleeding by statin use versus no statin use.** Important subgroups of patients in the EINSTEIN DVT and PE studies. ASA, acetylsalicylic acid; BMI, body mass index; CI, confidence interval; CrCl, creatinine clearance; HR, hazard ratio; VKA, vitamin K antagonist; VTE, venous thromboembolism.

For the treatment groups combined, 23 major bleeding events occurred during 778.8 patient*-*years (3.0 per 100 patient-years) with statin use compared with 89 during 3884.2 patient*-*years (2.3 per 100 patient-years) without use of statins, for an adjusted HR of 0.77 (95% CI 0.46–1.29). Due to the adjustments in the Cox regression model, the direction of this HR is in contrast to the crude comparison of rates by patient-years. This is largely because, in general, statin users are older and most of the major bleeding events in statin users occurred in the ≥60 years age group. Hazard ratios for major bleeding were similar for concomitant use of rivaroxaban-statin and enoxaparin/VKA-statin (p_interaction_=0.77, Figure 2). Hazard ratios for major bleeding with the use of statin were similar in all other important subgroups of patients (Figure 2).

### Extended treatment study

#### Recurrent venous thromboembolism

In the rivaroxaban group, no recurrent VTE occurred during 66.2 patient-years (0 per 100 patient-years) with statin use compared with 4 during 249.7 patient-years (1.6 per 100 patient-years) without use of statins (no HR calculated due to zero events). In the placebo group, 7 recurrent VTEs occurred during 57.3 patient-years (12.2 per 100 patient-years) with statin use compared with 33 during 249.9 patient-years (13.2 per 100 patient-years) without use of statins, for an adjusted HR of 0.81 (95% CI 0.35–1.86).

### Additional findings

A total of 40 cardiovascular ischemic events occurred in the statin users group, of which 15 occurred in the rivaroxaban group and 25 in the comparator group.

## Discussion

This secondary analysis of the EINSTEIN VTE treatment program showed that the use of concomitant statin therapy resulted in a moderate but non-statistically significant reduction in the rate of recurrent VTE in patients receiving anticoagulants or placebo. Major bleeding was numerically more common in patients receiving statin therapy but the adjusted hazard ratio showed a modest non-significant reduction; a phenomenon largely caused by a substantial age difference between statin users and non-users.

Interest in the possible pleiotropic effects of statins on VTE is not new [11, 12]. In the early 2000s, retrospective cohort and case control studies investigating the potential of statins to prevent VTE appeared in the literature [11, 13, 14]. The first randomized trial showed a 50% reduction in VTE risk in statin users versus non-users [15]. Subsequently, in the large randomized JUPITER trial in apparently healthy subjects at low risk of cardiovascular disease, rosuvastatin reduced the risk of VTE by more than 40% versus placebo [16]. However, a meta-analysis of 22 randomized trials with over 100,000 patients, comparing statins versus control in primary VTE prevention, suggested only a modest overall treatment effect with any statin (OR 0.89; 95% CI 0.78–1.01), although a subgroup analysis by statin suggested that rosuvastatin may be the most effective statin (OR 0.60; 95% CI 0.39–0.92) [4].

Evaluation of statins for secondary VTE prevention has only been done in observational studies and has shown similar results to our study. In two Danish population-based registries among patients with a hospital diagnosis of VTE, statin use was associated with a significantly lower risk of a recurrent VTE (adjusted HR 0.74; 95% CI 0.68–0.80 and 0.72; 95% CI 0.59–0.88, respectively) for recurrent VTE, compared with no statin use [5]. In a recent Dutch population-based registry of pharmacy records linked with hospital discharge records of patients hospitalized with an acute episode of PE, concomitant treatment with statins was associated with a reduced risk of recurrent PE (HR 0.50; 95% CI 0.36–0.70) [7]. This effect was observed both during and after stopping anticoagulant therapy. In addition, a dose–response relationship was shown for potency of statin therapy. The registries are limited by their retrospective assembly of cases and controls and the absence of standardized outcome assessments.

Some methodological aspects of our analysis warrant comment. First, the analyses of efficacy and safety with the use of statins were not pre-specified in the protocol or statistical analysis plan and there was no guidance in the protocol on the use of statins. Second, there were large differences in age, presentation of index event, co-morbidity and ASA use between the patients who used and did not use statins. Hence, all calculations of the relative effects of statins were adjusted for these important variables. Nevertheless, residual confounding is possible because it cannot be guaranteed that all underlying confounding factors could be considered in the models. We believe that the differences in age, co-morbidity and ASA use are based on the indication for statin use. More surprisingly was the difference in the distribution of the presentation of index event with an excess of PE in statin users. An explanation for this difference could be that patients who use statins are more likely to be evaluated for PE when they present to an emergency room with shortness of breath or thoracic pain. An alternative explanation could be based on the well described anti-inflammatory properties of statins [17–21], which may limit pain and other inflammatory symptoms that usually lead to a suspicion of DVT and presentation to the emergency room. Third, the literature supports a potentially higher risk of venous thromboembolic disease in patients with concomitant atherosclerotic disease [22–24]. Therefore, the observed hazard ratio could be an underestimation although we adjusted for cardiovascular co-morbidity. Finally, the power of the current analyses, including the dose–response analysis, was limited because only 1509 (18.3%) patients used statins. Actually, to detect a reduction in recurrent VTE of 24%, the overall effect observed in our analysis, a suitably powered 1:1 randomized study should include over 17,000 patients per group.

Our analyses have the following strengths: the use of statin medication was prospectively collected, intensively monitored, and centrally coded. Recurrent VTE and bleeding events were prospectively collected as well, and were reported using standardized forms. In addition, these events were adjudicated centrally using internationally accepted criteria without knowledge of treatment assignment and use of concomitant medication.

## Conclusions

Statins are simple to use, inexpensive, very safe and do not lead to bleeding. Therefore, the potential effect on recurrent VTE, which is in the range of that of ASA, deserves evaluation in a large randomized trial.

## Abbreviations

ASA: Acetylsalicylic acid
CI: Confidence interval
DVT: Deep vein thrombosis
HR: Hazard ratio
OR: Odds ratio
PE: Pulmonary embolism
VKA: Vitamin K antagonist
VTE: Venous thromboembolism.
